# Music interventions in 132 healthy older adults enhance cerebellar grey matter and auditory working memory, despite general brain atrophy

**DOI:** 10.1016/j.ynirp.2023.100166

**Published:** 2023-03-23

**Authors:** Damien Marie, Cécile A.H. Müller, Eckart Altenmüller, Dimitri Van De Ville, Kristin Jünemann, Daniel S. Scholz, Tillmann H.C. Krüger, Florian Worschech, Matthias Kliegel, Christopher Sinke, Clara E. James

**Affiliations:** a Geneva Musical Minds Lab, Geneva School of Health Sciences, University of Applied Sciences and Arts Western Switzerland (HES-SO), Geneva, Switzerland; b Faculty of Psychology and Educational Sciences, University of Geneva (UNIGE), Geneva, Switzerland; c Swiss Center for Affective Sciences, University of Geneva, Geneva, Switzerland; d CIBM Center for Biomedical Imaging, MRI HUG-UNIGE, Geneva, Switzerland; e Institute of Music Physiology and Musicians' Medicine, Hanover University of Music, Drama and Media, Hanover, Germany; f Center for Systems Neuroscience, Hanover, Germany; g Neuro-X Institute, Ecole Polytechnique Fédérale de Lausanne (EPFL), Campus Biotech, Geneva, Switzerland; h Faculty of Medicine, University of Geneva, Campus Biotech, Geneva, Switzerland; i CIBM Center for Biomedical Imaging, MRI EPFL & SP UNIGE-EPFL, Geneva, Switzerland; j Division of Clinical Psychology & Sexual Medicine, Department of Psychiatry, Social Psychiatry and Psychotherapy, Hanover Medical School, Hanover, Germany; k Center for the Interdisciplinary Study of Gerontology and Vulnerability, University of Geneva, Geneva, Switzerland

**Keywords:** Brain plasticity, Working memory, Atrophy, Grey matter, Music, Aging

## Abstract

Normal aging is associated with brain atrophy and cognitive decline. Working memory, involved in cognitive functioning and daily living, is particularly affected. Music training gained momentum in research on brain plasticity and possible transfer effects of interventions on working memory, especially in the context of healthy aging. This longitudinal voxel-based morphometry study evaluated effects of 6-month music interventions on grey matter volume plasticity and auditory working memory performance in 132 healthy older adults. This study is part of a randomized controlled trial comparing two interventions: piano practice (experimental group) and musical culture (musical listening awareness, active control). We report significant grey matter volume increase at whole-brain level in the caudate nucleus, Rolandic operculum and inferior cerebellum when merging both groups, but no group differences. Cerebellar grey matter increase, training intensity metrics and sleep were positively associated with tonal working memory improvement. Digit Span Backward verbal working memory performance also increased. Using region of interest analyses, we showed a group difference in the right primary auditory cortex grey matter volume, decreasing in the musical group while staying stable in the piano group. In contrast, a significant 6-month whole-brain atrophy pattern consistent with longer-term investigations of the aging brain was revealed. We argue that education for seniors should become a major policy priority in the framework of healthy aging, to promote brain plasticity and cognitive reserve, through stimulating group interventions such as music-making and active listening.

## Highlights

• A randomized control trial testing the effect of music on cognitive and brain aging.
• 6-month music training led to robust increases in grey matter in healthy older adults.
• Transfer effect: auditory working memory performance improved after training.
• Cerebellar grey matter and training intensity relate to working memory increase.
• Despite training, a whole-brain atrophy pattern consistent with the literature.

## 1 Introduction

The number and proportion of people over 60 years is growing at an unprecedented pace (UN, 2022; WHO, 2020). Therefore, efficient policies are mandatory to foster healthy aging (Abud et al., 2022), supporting functional abilities, independence, and well-being, particularly concerning the dementia threat. Age is the strongest risk factor for dementia (Daviglus et al., 2010). Yet, healthy aging, i.e., staying physically, socially, and cognitively active in combination with optimal management of health risk factors, like cutting alcohol or sugar, may delay dementia onset (Livingston et al., 2020; WHO, 2021). Healthy aging is a plurifactorial process arising from the interaction of inevitable biological decline, the stimulation of individual capacities (eg. cognitive or physical capacities) and physical/social environmental factors (WHO, 2021). Stimulating group interventions are promising approaches to support healthy aging (Intzandt et al., 2021). The present applied cognitive neuroscience study fits in this framework, reporting on a randomized controlled trial examining the effects of two music interventions on cognitive and brain health in musically naïve older adults.

Brain atrophy is a typical phenomenon of normal aging. One year is sufficient to detect significant global atrophy (Fjell et al., 2009), distributed in an anterio-posterior gradient, with faster rates of decline in the frontal and temporal-parietal cortex (Bagarinao et al., 2022; Fjell and Walhovd, 2010; Fjell et al., 2009). Especially the prefrontal cortex, hippocampus, striatum and cerebellum show rapid decline (Fjell and Walhovd, 2010; Fraser et al., 2015; Hicks et al., 2022; Jiang et al., 2014). The thalamus (Jiang et al., 2014; Pfefferbaum et al., 2013; Raz et al., 2010) and Heschl's gyrus (Crivello et al., 2014; Jiang et al., 2014; Profant et al., 2014) are also particularly affected by aging. Yet, to the best of our knowledge, no investigation evaluated atrophy over a shorter period than one year. More fine-tuned longitudinal investigations are needed to reach a consensus on the differential local rate of atrophy (Raz et al., 2010) and the differences between associated normal and pathological cognitive decline (Desgranges et al., 2008; Fjell and Walhovd, 2010; Fotenos et al., 2005; Persson et al., 2012). Finally, there is a lack of data on the potential countereffects of healthy aging interventions on atrophy.

Normal aging impacts not only brain structure, but also its functions and associated behavior. Executive functions and working memory (WM) suffer most (Buckner, 2004; Ferguson et al., 2021; Rozas et al., 2008). Core executive functions include initiation, shifting, monitoring, inhibition and WM, extending to complex behavior like abstract reasoning and planning (Diamond, 2013; Miyake and Friedman, 2012). WM involves temporarily storing, updating and manipulating information while no longer perceptible, scaffolding complex tasks such as abstract reasoning or planning (Baddeley, 2010; Dehn, 2017; Miller et al., 2018). Moreover, WM is involved in many aspects of cognitive functioning comprising for instance language and music (Gathercole and Baddeley, 2014; Schulze and Koelsch, 2012), visuospatial orientation (Coluccia, 2008), but also, activities of daily living (Cantarella et al., 2017; McDougall et al., 2019), crucial for independence and well-being (Na and Streim, 2017).

Functional Magnetic Resonance Imaging (MRI) shows that WM is supported by the same brain regions prominently impacted by age-related atrophy, namely a frontoparietal network, including the striatum (Eriksson et al., 2015b). Additional regions, like the medial temporal lobe, auditory cortex and cerebellum, are involved depending on task requirements (e.g., maintenance or further manipulation), sensory input (e.g., auditory/verbal or visual/object), and stages of processing (encoding, maintenance, or response) (Chai et al., 2018; Christophel et al., 2017; Deldar et al., 2021; Emch et al., 2019; Eriksson et al., 2015a; Rottschy et al., 2012; Wager and Smith, 2003; Yaple et al., 2019). Meta-analytic reports on the age-related functional alteration of WM highlight prefrontal cortex activity suppression (Yaple et al., 2019), or differential changes in activity depending on prefrontal subregions (increase vs. decrease, Rajah and D'Esposito, 2005). Such brain activity changes relate to WM load (Spreng et al., 2010) and performance (Evangelista et al., 2021). Only a few studies specifically investigated relationships between Grey Matter (GM) atrophy and WM performance in healthy older adults (Fjell and Walhovd, 2010; Salthouse, 2011). Age-related decline in WM may be mediated by decreased volume of lateral prefrontal cortex or its subregions (Gunning-Dixon and Raz, 2003; Head et al., 2009; Kennedy et al., 2009; Salat et al., 2002). Yet most of those studies focusing on the prefrontal cortex were cross-sectional. More longitudinal investigations are needed on the relationships between normal aging, brain structure, and WM at the exploratory whole-brain level and/or based on a priori hypothesis testing. For instance, the auditory cortex and the cerebellum (Bernard and Seidler, 2013; Marvel and Desmond, 2010) are regions of interest in the specific context of auditory WM (Kumar et al., 2016; Yu et al., 2021).

Beyond the detrimental effects of age-related decline, brain and cognition may also change positively as a function of experience over the whole lifespan. The aging brain retains considerable plasticity (Boyke et al., 2008; Grady, 2012), relating to cognitive reserve (Esiri and Chance, 2012; Stern, 2012) – the ability to cope with damage and decline. Music training has gained a lot of momentum as a model in the research on brain plasticity driven by learning (Altenmüller and Schlaug, 2015; Jancke, 2009; Münte et al., 2002; Rus-Oswald et al., 2022; Vuust et al., 2022; Wan and Schlaug, 2010; Zatorre et al., 2012), but also on behavioral transfer (Besson et al., 2011; Bigand and Tillmann, 2022; James et al., 2020b; Martins et al., 2021; Wang, 2022) and more recently, to the mediation of cognitive decline in the healthy aging framework (Bugos, 2018; Galinha et al., 2020; James et al., 2020a; Jünemann et al., 2022; Rouse et al., 2022; Schneider et al., 2018; Worschech et al., 2021; Worschech et al., 2022). Music making constitutes an excellent model of plasticity and transfer effects, because learning to play an instrument is a cross-modal activity, eliciting not only the closely related sensorimotor domains (close or near transfer) but also more distant ones (far transfer), for instance processing speed, affective domains, memory, language, executive function or abstract reasoning (James, 2012; James et al., 2020b; Jäncke, 2012; Koelsch, 2011; Oechslin et al., 2013a; Wan and Schlaug, 2010), etc. The affective and rewarding aspects of musical activities offer an intrinsic incentive, supported by neurochemistry that may reinforce learning (Chanda and Levitin, 2013; Ferreri et al., 2019).

Cross-sectional evaluation of cerebral differences between young adults with different degrees of musical expertise (non-musicians, amateur and expert pianists) revealed gradual changes in brain structure and function and higher-order cognition according to training intensity and expertness. Progressive plasticity as a function of musical expertise occurred in the fusiform gyrus, sensorimotor areas, hippocampus, prefrontal and anterior insular cortex and in the bilateral posterior cerebellum (James et al., 2014a; Oechslin et al., 2013a; Oechslin et al., 2017; Oechslin et al., 2018; Oechslin et al., 2013b) including larger GM volume in Heschl's gyrus (Dalboni da Rocha et al., 2020; Gaser and Schlaug, 2003; James et al., 2014a; Schneider et al., 2002). At the behavioral level, music training transfers to WM abilities, numerous studies reporting enhanced auditory/visual WM skills in musicians as compared to non-musicians (James et al., 2020b; Oechslin et al., 2013c; Schulze and Koelsch, 2012; Yurgil et al., 2020) including healthy older adults (Mansens et al., 2018; Schneider et al., 2018). Interestingly, some studies highlighted a protective association between lifelong music instrumental practice and cognitive impairment/dementia risk or delayed onset (Balbag et al., 2014; Gooding et al., 2014; Verghese et al., 2003), suggesting that music training may contribute to cognitive reserve (Cabeza et al., 2018). Music making may thus promote preservation of WM against cognitive decline or provide additional resources that may mitigate loss in WM performance.

Such a hypothesis is supported by a few longitudinal investigations conducted in healthy older adults, showing enhanced WM performance following music interventions (Bugos, 2019; Bugos et al., 2007; Bugos and Wang, 2022; Dege and Kerkovius, 2018; but see Seinfeld et al., 2013). However, training only lasted around 15–16 weeks except for one study running over 6 months (Bugos et al., 2007). While randomization was carried out in almost all studies, the sample size was often small (mean = 25.7 participants per group, with a range of 8–49 participants in the musical group). Only one study included music listening as an active control condition for a piano intervention (Bugos, 2019), reporting significant cognitive benefits only in the piano practice group. In absence of an appropriate active control group, WM transfer effects detected in other studies may not be attributed to instrumental practice per se but to mere listening or social interaction. Moreover, as relationships between gain in WM performance and musical training intensity (eg. number of lessons followed or time spent on homework) were not evaluated, test-retest effects and/or sampling issues can not be ruled out, even though group differences were detected. In addition, effects reported mostly concerned visual WM, sometimes embedded in tests also evaluating other domains, such as processing speed and visuo-spatial abilities, planning and inhibition. Significant results may thus arise from improvements in the latter cognitive domains but not from WM progress. Two studies included the Backward Digit span in their evaluation, a WM test provided orally, but reported no group difference (Dege and Kerkovius, 2018; Seinfeld et al., 2013). Yet, one may expect music training to convey larger transfer effects in the auditory than in the visual WM domain. Finally, none of these investigations integrated neuroimaging into their protocol nor relationships to intervention intensity. Thus, neither brain plasticity associated with musical training effects on WM, nor the potential link between atrophy and cognitive performance were examined in healthy older adults.

While Heschl's gyrus, hosting the primary auditory cortex (Marie et al., 2015), is particularly affected by age-related atrophy as mentioned above (Crivello et al., 2014; Jiang et al., 2014; Profant et al., 2014), the grey matter encompassing this region is also widely associated with music expertise/training-induced plasticity effects on the left, right or both hemispheres in children, adults and older adults (Bermudez et al., 2009; Dalboni da Rocha et al., 2020; Gaser and Schlaug, 2003; James et al., 2014a; Schneider et al., 2002; Worschech et al., 2022). Yet no investigation evaluated in vivo primary auditory GM volume plasticity following music training. The primary auditory cortex is a highly granular cortex with a prominent layer IV, which is the principal target for thalamic auditory input. Thus, we may expect that musical training plasticity effects, related to increased auditory processing and WM, may countervail in part age-related atrophy in primary auditory subregions in one or both hemispheres. In particular, we expect larger plasticity effects in the piano group compared to the musical culture group in relation to auditory WM advantages.

The present study evaluated brain and behavioral changes in 132 musically naïve healthy older adults after 6 months of music interventions. We examined the following brain measures and their relationships to behavior over time and between groups (experimental group: piano, N = 66; active control group: musical culture, N = 66). First, GM volume increase at the whole-brain and the primary auditory cortex level; Second, auditory WM performance changes (positive transfer, increase); Third, positive relationships between GM plasticity, training intensity metrics (eg. number of lessons followed, homework) and WM performance; Finally, GM atrophy and group differences in relation with WM performance. Although musical culture intervention might provide benefits, we expected larger plasticity effects, WM improvement, and lower atrophy in the piano group compared to the musical culture group (active listening). Learning how to play a musical instrument is a very complex task. WM, cognitive load, generation of predictions (predictive coding of actions and sounds, Vuust et al., 2022), flexibility, pitch analysis, sensorimotor activity, and interplay with other cognitive domains, may be lower when listening to music than when making it, which may be reflected at the behavioral and the brain plasticity level.

## 2 Material and methods

### 2.1 Randomized controlled trial

This study is part of a randomized controlled trial protocol, testing the benefits of piano instruction (P, experimental group) against musical culture (MC, musical listening awareness without practice, active control group), and described elsewhere (James et al., 2020a). Briefly, 155 healthy, non-musician, older adults enrolled in this interventional multisite study conducted in Germany and Switzerland (62–78 years old; 92 females; 63 individuals in Geneva and 92 in Hanover). Inclusion criteria included overall good physical/mental health, right-handedness, retirement, non-reliance on hearing aids, and importantly, less than 6 months of formal musical training over the lifespan. We evaluated global cognitive functioning using the Cognitive Telephone Screening Instrument (COGTEL, assessing verbal short- and long-term memory, WM, verbal fluency, inductive reasoning, prospective memory, Ihle et al., 2017; Kliegel et al., 2007) to exclude participants with potential dementia onset (cut-off score of 10, Ihle et al., 2017). COGTEL score, age, gender, and education level (1–6) were entered in the stratified randomization procedure (see James et al., 2020a), ensuring equal distribution of the participants according to these four factors into the two groups (see Table 1, no differences between both groups occurred).

Participants received 12 months of interventions consisting of 1-h weekly music training. They were instructed to practice at least 30 min at home 5 days a week (homework), and to record the duration of homework per day and per week. The content of both interventions is described in detail elsewhere (James et al., 2020a; Jünemann et al., 2022; Worschech et al., 2022; Worschech et al., 2021). Briefly, Piano (P) lessons were provided in dyads. Participants learned how to perform pieces with both hands while adopting a correct position, play with various expressions, read a musical score and improvise. They also practiced rhythm exercises. Musical culture (MC) lessons included 4–6 participants. Those lessons entrained analytical music listening, from basic music properties, like perceiving different instruments, to more theoretical concepts, including various musical styles and conveyed emotions as well as music history (epochs, genres, etc.). The role of the active control group was to allow controlling for enjoyment, social, contextual, and learning effects not related to musical practice. Any type of music production was avoided (e.g. clapping, singing, etc.).

Data was collected at 4 timepoints (T0 = baseline, T1 = 6 months, T2 = 12 months, T3 = 18 months i.e. 6 months post-intervention) with psychometric testing on cognitive and perceptual-motor aptitudes as well as via wide-ranging functional and structural neuroimaging and blood sampling (James et al., 2020a). Group attribution was revealed after baseline testing (T0).

## 3 Participants

Here we present the results of analyses conducted on 132 individuals over 6 months, i.e. from T0 to T1 (see Table 1 for demographics). Only 137 out of the 155 participants went through the MRI data collection for compatibility/safety reasons. In addition, five participants were excluded for image quality reasons (see Image acquisition and Preprocessing). Final sample size is thus 132 individuals. Randomization factors distribution did not differ between the two groups of 66 individuals (Table 1).

### 3.1 Data acquisition

#### 3.1.1 Image acquisition and pre-processing

Magnetic Resonance Images were acquired on 3.0 T Siemens MRI scanners (Hannover Medical School: Magnetom Skyra; Geneva Medical University Center, Brain and Behaviour Laboratory: Magnetom Tim Trio; Siemens, Erlangen, Germany) using 32-channel head coils. Both sites used identical scanning parameters. A high-resolution T1-weighted structural image was acquired using an MP2RAGE sequence (Marques et al., 2010, duration = 8:22 min, voxel size = 1 mm^3^ isotropic; 176 slices, FOV = 256x240 × 176 mm; TR/TE = 5000/2.98 ms; TI1/TI2 = 700/2500 ms; flip angle 1/2 = 4°/5°).

MRI preprocessing was performed using the statistical parametric mapping software (SPM12, Wellcome Department of Imaging Neuroscience, London, UK, https://www.fil.ion.ucl.ac.uk/spm/) and the Computational Anatomy Toolbox (CAT12, version 12.7 r1742, https://neuro-jena.github.io/cat/). GM volume maps were extracted following the Voxel-Based Morphometry (VBM) protocol ( Ashburner and Friston, 2000) implemented in the longitudinal data preprocessing pipeline of CAT12 (Gaser et al., 2022; Gaser and Kurth, 2017). CAT12 is optimal for longitudinal VBM because it is fully automatized, and it allows detecting subtle and robust effects over time. The strength of CAT12 longitudinal preprocessing resides in the joint registration of the longitudinal individual images to MNI space and the maintenance of their temporal coherence by processing the longitudinal data for each subject at each timepoint simultaneously. Longitudinal data analysis requires customized processing, as a voxel- or point-wise comparability needs to be assured not only across subjects, but also across timepoints within subjects. This requires an inverse-consistent (or “symmetric”) realignment as well as an intra-subject bias field correction. CAT12 realigned the (individual) images from all time points using inverse-consistent rigid-body registrations and applied intra-subject bias-field correction. Subsequently, the resulting images were processed individually using the standard pipeline. The spatial adaptive non-local means denoising filter was applied to correct for noise. Images were then automatically segmented into GM, white matter and cerebrospinal fluid using the tissue probability map provided in SPM (non-brain structures were removed, eg. skull bones). As a result of the segmentation, the Total Intracranial Volume (TIV) and its components (GM, white matter and cerebrospinal fluid volumes) were automatically computed for each individual. The segmented images were normalized to the standard Montreal Neurological Institute (MNI) space using DARTEL (Diffeomorphic Anatomical Registration using Exponentiated Lie algebra, Ashburner, 2007). A mean transformation for all time points was calculated and applied to all individual segmented images to correct for global brain shape differences yet keep as much intra-individual variability over time as possible. Images were then modulated based on Jacobian determinants. Finally, GM volume maps were smoothed with an isotropic Gaussian kernel size of 8-mm full width at half maximum (FWHM). In addition, images were statistically checked for homogeneity and overall quality (CAT12 retrospective quality assurance framework), which led to the exclusion of five outliers due to artifacts mostly related to movements. This is especially relevant for longitudinal studies since systematic differences in noise, artifact, or inhomogeneity between scans obtained over time can be confounded with the training effect of interest (Marie and Golestani, 2016).

#### 3.1.2 Behavioral data

The psychometric battery included 15 tests (see James et al., 2020a for a full description) covering different cognitive and sensorimotor domains as well as questionnaires about quality of life and physical activity. The order of the tests was (pseudo) randomized between individuals and timepoints. Hereafter we describe the tests and data relevant for this VBM study on WM.

#### 3.1.3 Intervention intensity metrics and sleep

Presences and absences of participants were recorded over the intervention to compute the number of lessons that were followed by each individual over 6 months (T0 to T1). In addition, participants were requested to work at least 30 min per day on their homework and report their homework time weekly. As part of the extensive pre-screening demographic and health questionnaire, the average sleep duration in hours at baseline (subjective report) was inventoried, because the variability of sleep duration impacts executive function (Lowe et al., 2017). Moreover, lack of sleep interferes with WM performance (del Angel et al., 2015; Diamond, 2013; Lücke et al., 2021) and learning (Diekelmann and Born, 2010).

#### 3.1.4 Tonal working memory task: easy condition

**Fig. 1:**
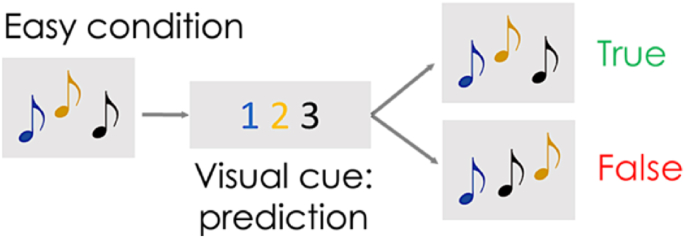
**Tonal working memory task: schema of the events characterizing one trial of the easy condition.** From left to right, first, the participant hears a 3-tone pattern. Second, a visual cue indicating 1-2-3 appears. This cue predicts no change in the order of the tones between the first tone pattern and the following tone pattern. Third, the participant hears this second 3-tones pattern with the very same tones, either presented in the same order (true) or in a different order (false). Finally, the participant indicates whether the prediction of the visual cue was true or false.

Participants performed a tonal WM task in the MRI scanner to evaluate tonal WM networks as part of the functional MRI session. The task was adapted from Malinovitch et al. (2017). This task includes three conditions that alternate randomly, a control, easy and difficult condition with different WM loads (18 trials per condition). During this task, the participant hears two tone patterns (3 beeps of sinus tones, 1 beep/tone = 0.4 s) separated by a visual cue predicting a potential change in the order of the notes between pattern 1 and pattern 2 (Fig. 1). The cue consists of a series of numbers, referring to the order of the tones (eg. 1-2-3 means that the order of the tones will not change between the pattern 1 and 2 while 3-2-1 indicates that the tone pattern will reverse). Participants used a two-button response pad to confirm or infirm the prediction (true or false). The cue also indicates the condition.

During the easy condition trials (Fig. 1), the prediction always indicates an absence of change in the order of the notes (cue:1-2-3) between the two 3-tone patterns. If the tone pattern does not change (pattern 1 = pattern 2), the participant must indicate that the prediction was true. If the order of the tones changes, he/she should indicate that the prediction was false. This condition elicits tonal WM, particularly the updating component, but also saliency and attention as the participant must isolate the tonal patterns from the MRI noise, which is not an easy process.

In the current study, we only explore the training effects on tonal working memory performance associated with this easy condition because the participants failed the difficult condition. The difficult condition accuracy only reached chance-level (50.8 ± 14.1%). Briefly, the cue associated with the difficult condition is a prediction of a change (eg. 3-2-1, meaning the pattern will be reversed, or 2-1-3, meaning the first two tones will be inversed but the third tone will stay at the same position). The participant must manipulate the tones of the first pattern using tonal mental imaging, to determine if a change occurred in second pattern. Finally, there was no prediction in the control condition that was associated with 1-1-1, 2-2-2, or 3-3-3 cues and repetitive tones patterns (three tones of the same frequency in both patterns). The participant simply had to push an indicated button, which did not engage working memory per se but served as a baseline for functional MRI contrast analyses.

#### 3.1.5 Verbal WM: Backward Digit Span

Participants performed the Digit Span task as implemented in the Wechsler Adult Intelligence Scale (Wechsler, 2011) as part of the psychometric battery. This task requires participants to repeat a series of digits of increasing length. Numbers are repeated in the same order as read aloud by the examiner for the Digit Span Forward, while they should be repeated in reverse order of that presented by the examiner in the Digit Span Backward. Here we will focus on the Backward Digit Span which can be interpreted as a measure of verbal WM (Pisoni and Cleary, 2003). During this task one must memorize, update, and manipulate the numbers to be able to reproduce the given target list with the items presented in reverse order.

### 3.2 Statistical analysis

Statistics were performed with SPM12 (v7771), MATLAB R2018a (v9.4.0.949201) and JMP Pro (v15.2.1).

#### 3.2.1 Demographics and randomization

To assess our randomization process (see previous section, Randomized controlled trial), and the effect of sampling (only 132 individuals over 155 could be analyzed in this study as described in the Participants section), we provide descriptive statistics (Table 1) of the sample analyzed here and its constitution, the two groups of 66 individuals. Group comparisons for Age, Gender, Education and the COGTEL score were conducted. After evaluating normality with Shapiro-Wilk tests, statistical testing was performed using an unpaired two-sample Wilcoxon test for Age, a two-sample independent *t*-test for COGTEL score, and chi-squared tests for gender and education.

#### 3.2.2 VBM analyses and grey matter volume extraction

Images difference contrasts between timepoints were generated for each subject (T1-T0 and T0-T1, T0: baseline MRI, T1: 6 months MRI) using the ImCalc function of SPM. The resulting images were then used as input for whole-brain statistics. In all whole-brain analyses, we controlled for the confounding factors of age, TIV, and site (Geneva or Hanover). TIV was added to correct for brain size differences. This image difference contrast approach was selected to account for fixed within-subject model components, such as the site covariate. All analyses included an inclusive GM mask to restrict the analysis to this tissue only (SPM GM tissue probability map binarized with an intensity threshold > 0.15) and avoid possible edge effects around the border between grey and white matter. We present results following a statistical threshold of *p* < 0.001 (uncorrected for multiple comparisons, UNC) and *p* < 0.05 Family-Wise Error corrected (FWEcorr) with a minimum cluster size of 20 voxels (*k*). Uncorrected results are presented to give a broader perspective as FWEcorr is very restrictive. To evaluate group differences in GM change, we performed a two-sample *t*-test in SPM. In the absence of a group effect, we then ran a voxel-wise one-sample *t*-test assessing overall Grey Matter volume (GM vol) change over time in the entire sample (132 individuals, both groups). This model allowed to evaluate both GM volume increase, and GM volume decrease or atrophy, over 6 months.

To evaluate relationships between VBM and behavioral data, GM volume from significant clusters were extracted using the SPM get_totals function (GM volume increase: 4 clusters at *p* < 0.05 FWE; GM volume decrease: 6 clusters > 300 voxels at *p* < 0.05 FWE). No absolute intensity threshold was applied for extraction. In addition, for a priori hypothesis testing, GM volume was extracted in left and right primary auditory cortex Regions Of Interest (ROI). 6 binary ROIs were created from the Jülich brain atlas (see Primary auditory cortex subdivisions: left and right TE1.0, TE1.1 and TE1.2 areas), and GM volume was extracted using SPM get_totals function, binary ROIs as masks of extraction, and T0 and T1 individual smoothed GM volume maps as input. Anatomical labeling of the peaks was conducted based on visual inspection, neuroanatomical knowledge and AAL atlas (Tzourio-Mazoyer et al., 2002).

#### 3.2.3 Tonal working memory performance and grey matter volume increase

To evaluate whether there is a significant improvement in the tonal WM performance over time and between groups, a 2x2 repeated-measure Analysis of Covariance (ANCOVA) model was performed, including the repeated within-subject factor time (T0 and T1 tonal WM accuracy) and between-subject factor group (piano and musical culture). Normality was evaluated beforehand with Shapiro-Wilk tests.

The Time factor being significant but not the group factor, a post-hoc *t*-test of the tonal WM performance mean difference (raw data, T1 - T0 accuracy) as compared to 0 was performed in all participants, both groups combined, to characterize such Time effect, and separately in each group for descriptive purposes.

In this 2x2 repeated-measure ANCOVA model, training intensity (number of music lessons followed over 6 months and average time spent on homework in minutes per week over 6 months), sleep amount (subjective report of the average duration of sleep in hours per night at baseline), and significant GM volume regional increases were included as covariates of interest. Specifically, individual GM volume changes were extracted for each of the four clusters showing a significant increase of GM volume at *p* < 0.05 FWE: T1 – T0 left caudate, left cerebellum, right cerebellum, and right Rolandic Operculum GM volumes. Finally, age at baseline, Site (1 = Geneva, 2 = Hannover) and Total Intracranial Volume were included as nuisance factors in the model. In total, 126 individuals performed the tonal WM task. This model includes only 112 individuals over 126 as some participants did not correctly report homework and sleep data. Yet, taking out homework and sleep variables do not change the main results (significant Time effect and Time*Number of lessons followed over 6 months and Time*L*R cerebellum interactions).

A post-hoc ANCOVA model on the tonal WM progress (T1-T0 tonal WM accuracy) was carried out to evaluate the percentage of variance explained by the combination of significant factors. Post-hoc linear regressions were then computed to understand the effects of the total number of lessons over 6 months, the average time spent on homework per week at T1 and the average amount of sleep per night at T0. For these linear regressions, tonal WM performance progress (y-axis) was adjusted by all factors except the effect of interest for the plot (x-axis factor). Further linear regressions were carried out to evaluate the triple interaction Time*L cerebellum*R cerebellum GM volume change. To achieve this goal, we categorized participants according to their GM volume pattern of change, each side separately (left cerebellum, 2 categories: N = 54 with GM volume increase i.e., positive GM values, N = 58 with GM volume decrease i.e., negative values; right cerebellum, 2 categories: N = 57 with GM volume increase, N = 55 with GM volume decrease).

Finally, as a complementary analysis, we evaluated whether the total GM volume change in the left and right cerebellum combined (sum of the left and right volume change over time: T1-T0 left cerebellum volume + T1-T0 right cerebellum volume) linearly predicted tonal WM performance progress. In the above-mentioned post-hoc analyses, cerebellar GM volumes were adjusted for total intracranial volume to control for head size differences.

#### 3.2.4 Verbal working memory (Digit Span Backward)

To evaluate whether there is a significant improvement in the Backward Digit Span verbal WM performance over time and between groups, the same statistical method was applied than for the tonal WM performance. A 2x2 repeated-measure ANCOVA model was performed, including the repeated within-subject factor time (T0 and T1 Backward Digit Span score) and between-subject factor group (piano and musical culture). Here again, training intensity (number of music lessons followed over 6 months and average time spent on homework in minutes per week over 6 months), sleep amount (estimated average duration of sleep-in hours per night at baseline), and significant GM volume regional increases were included as covariates of interest (T1 – T0 left caudate, left cerebellum, right cerebellum, and right Rolandic Operculum GM volumes). Age at baseline, Site and Total Intracranial Volume were included as nuisance factors in the model. This model includes only 118 individuals over 132 as some participants did not correctly report homework and sleep data. Independently, the distribution of the T0 and T1 Digit Span Backward score did not follow the normal law, but their residuals from the 2x2 repeated-measure ANCOVA model did (Anderson-Darling normality test). Thus, we opted for non-parametric post-hoc testing.

Finally, the 2x2 ANCOVA including 12 factors, which is detrimental for statistical power, we re-evaluated the significance of the Digit Span verbal WM progress over time (T1 – T0 Backward Digit span score) using a simpler procedure, a Wilcoxon Signed-Rank test as compared to 0 in all participants as a complementary analysis, both groups combined, and separately in each group for descriptive purposes.

#### 3.2.5 Atrophy and auditory working memory performance

The above-mentioned models evaluating the relationships between GM volume plasticity and tonal/verbal WM performance were repeated with the addition of GM atrophy variables (FWE corrected total atrophy volume over 6 months or independent atrophy measurements in 6 regions surviving FWE correction and above 300 voxels).

### 3.3 Group difference in grey matter volume of the primary auditory cortex

#### 3.3.1 Primary auditory cortex: TE1.0, TE1.1, and TE1.2 areas (region of interest analyses)

**Fig. 2:**
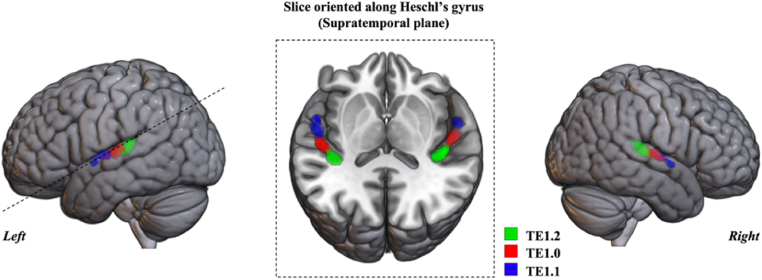
**Locations of the cytoarchitectonic subdivisions of the primary auditory cortex TE1 areas used as region of interest (ROI) for the grey matter volume group comparison analysis.** ROI extracted from the probabilistic cytoarchitectonic maps provided by the Jülich brain (Amunts et al., 2020) after applying a threshold of 40%. This threshold was selected upon careful inspection of neuroanatomy, being a good trade-off between ROI size, anterior/single Heschl's gyrus coverage, and probability to be in the primary auditory cortex. The dashed line indicates the cut performed along the Sylvian Fissure and Heschl's gyrus, in the supratemporal plane, to obtain the slice presented in the center of the image showing left and right colour coded TE1 areas (TE1.0 is in red, TE1.1 in blue and TE1.2 in green). (For interpretation of the references to color in this figure legend, the reader is referred to the Web version of this article.)

To test the hypothesis of a differential change over time in the left or right primary auditory cortex in favor of the piano practice as compared to the musical culture, we designed regions of interest (ROI) based on the Jülich brain's probabilistic observer-independent cytoarchitectonic maps (Fig. 2, primary auditory cortex regions of interest, Amunts et al., 2020; Morosan et al., 2001; Rademacher et al., 2001). Altogether areas TE1.0, TE1.1 and TE1.2 constitutes TE1 (Morosan et al., 2001; Rademacher et al., 2001). TE1 is equivalent to Brodmann area 41, Brodmann's cytoarchitectonics definition of the primary auditory cortex (A1) with highly granular cortex and a prominent layer IV, which is the principal target for thalamic auditory input. TE1.0 has the widest granular layer IV and is considered as the koniocortical core field (Morosan et al., 2001). Probabilistic maps of the three subdivisions of the primary auditory cortex (TE1.0, TE1.1, and TE1.2) were thresholded to 40% and converted to binary ROI using SPM ImCalc function. This threshold was selected upon visual inspection, being a good trade-off between probability, ROI size, and macroanatomy coverage (anterior/single Heschl's gyrus, Marie et al., 2015).

To evaluate whether there is a significant change in primary auditory GM volume over time and between groups, a 6x2 repeated-measure ANCOVA model was performed, including the repeated within-subject factor TE1 GM volume 6-month change (left T1-T0 TE1.0, left T1-T0 TE1.1, left T1-T0 TE1.2, right T1-T0 TE1.0, right T1-T0 TE1.1, right T1-T0 TE1.2) and between-subject factor group (piano and musical culture) in the 132 individuals. Site, Baseline Age and Total intracranial volume (TIV) were included as confounders.

Following the trend for a significant interaction a Group*ROI GM Change interaction and the ANCOVA Least Squares Means plot included in the analysis, clearly indicating that this trend might relate to the right TE1.0, an additional post-hoc ANCOVA model was designed to evaluate regional change between groups and over time (right T1-T0 TE1.0). Leverage residuals and least squares means were used to illustrate the significant effects. Post hoc testing of the raw mean as compared to 0 was performed using a *t*-test for the piano group and a Wilcoxon Signed-Rank test for the musical culture group to inform on raw group mean data and the significance of the change independently in each group. We added training intensity metrics to the model to evaluate if they contributed to the right right TE1.0 GM volume change over time (Number of lessons followed over 6 months and average time spent on homework per week at 6 months).

In a final step, we went back to our two models of interest evaluating auditory WM progress and added R T1-T0 TE1.0 GM volume as an additional factor to evaluate whether the plasticity of this primary auditory cortex subregion contributed to a change in the auditory WM performance.

## 4 Results

### 4.1 Demographic

**Table 1:** **Randomization factors demographics at T0 (mean ± standard deviation or percentages).** Group comparisons did not reveal any significant differences between both groups for age, gender, education level and COGTEL score. Group differences were evaluated using an unpaired two-sample Wilcoxon test for Age, a two-sample independent *t*-test for COGTEL score, and chi-squared tests for gender and education. Education is a categorical variable with 6 levels (1 = primary school, 2 = middle school, 3 = high school, 4 = Bachelor's degree, 5 = Master's degree, 6 = doctorate degree), COGTEL = Cognitive Telephone Screening Instrument, P = Piano group, MC = Musical Culture,♀ = female.

Randomization factors at T0 (mean ± standard deviation)				Group comparison	
Factors	Population (N = 132)	P (N = 66)	MC (N = 66)	statistic	*p*-value
Age	69.2 ± 3.5	69.2 ± 3.2	69.2 ± 3.8	*Z* = 0.58	0.56
Gender	77♀ (58%)	38 ♀ (58%)	39 ♀ (59%)	*χ*^*2*^ = 0.03	0.86
Education	3.9 ± 1.3	3.9 ± 1.4	4 ± 1.3	*χ*^*2*^ = 3.4	0.63
COGTEL	31.4 ± 7.2	31 ± 7.1	31.9 ± 7.3	*t* = −0.7	0.48

### 4.2 Whole-brain grey matter volume differences between P and MC groups

Group comparisons (two-sample *t*-test, P > MC and T1 > T0; MC > P and T1 > T0) did not reveal any statistically significant differences in GM volume increase over 6 months of training (*p* < 0.001, uncorrected, *k* = 20 voxels).

### 4.3 Whole-brain grey matter volume increase over 6 months in 132 participants

**Fig. 3:**
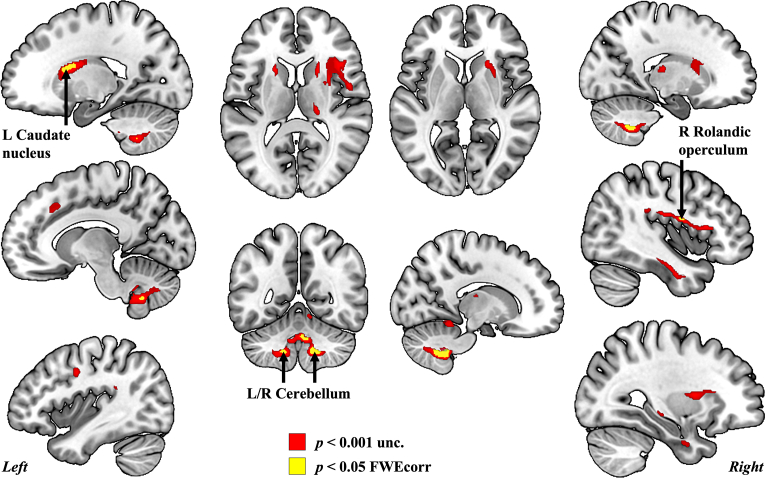
**Grey matter volume increase over 6 months in 132 individuals following music interventions (T1 > T0).** Results displayed on MNI152 brain template with an extent threshold of *k* = 20 voxels at *p* < 0.001 uncorrected for multiple comparisons in red and at *p* < 0.05 family-wise error corrected in yellow. Significant clusters at p < 0.05 FWE are indicated within the figure, they include the left and right cerebellum, the left caudate nucleus and the right Rolandic operculum (L: left; R: right). (For interpretation of the references to color in this figure legend, the reader is referred to the Web version of this article.)

The analysis of GM volume increase conducted in all participants, both music training groups combined, revealed a widespread pattern of positive GM volume changes over 6 months (Fig. 3, Supplementary Table 1, *p* < 0.001, uncorrected, *k* = 20 voxels, one-sample *t*-test) in the neocortex, subcortical structures, and in the cerebellum. Four clusters are significant after multiple comparison corrections (Fig. 3, Table 2, *p* < 0.05 FWE corrected, *k* = 20 voxels).

**Table 2:** **Locations of clusters at peak voxels and statistics associated with grey matter volume increases detected over 6 months of music training in all individuals at *p* < 0.05 Family-wise error corrected (N = 132, *k* = 20 voxels).** Four clusters are significant: left cerebellum, right cerebellum, left caudate nucleaus and right Rolandic operculum (L: left; R: right, inf: inferior; MNI: Montreal Neurological Institute; N = number).

Location of peak voxels	MNI coordinates (x, y, z)	Cluster size (N voxels)	T-value (peak-level)	FWEcorr *p*-value (peak-level)
R inf. cerebellum (VIII, IX)	15, −52, −45	285	6.79	*p* < 0.001
L inf. cerebellum (VIII, IX)	−12, −57, −46	68	5.67	*p* = 0.003
L caudate nucleus	−20, 10, 18	33	5.66	*p* = 0.003
R rolandic operculum	46, −2, 16	31	5.49	*p* = 0.007

### 4.4 Auditory working memory improvement over 6 months

#### 4.4.1 Tonal working memory progress is associated with bilateral cerebellar grey matter volume increase, training intensity metrics and sleep duration

The repeated-measure 2x2 ANCOVA model detected a significant effect of Time [*F*(1; 100) = 6.1, *p* = 0.02]. We observed a significant 5.7% raw gain of tonal WM performance over 6 months in all participants, both groups combined (post-hoc *t*-test of the mean compared to 0, T1– T0 tonal WM accuracy = 5.7 ± 0.19, *t* = 3.3, *p* = 0.001; mean T1– T0 tonal WM accuracy in the P group = 6.4 ± 0.2, *t* = 2.5, *p* = 0.007; mean T1– T0 tonal WM accuracy in the MC group = 4.9 ± 0.19, *t* = 2.1, *p* = 0.04; two-tailed *t* tests).

This ANCOVA model showed that training intensity, sleep amount, and GM volume plasticity factors influenced this gain of performance The following significant interactions were found: Time * Number of lessons over 6 months [*F*(1; 100) = 10, *p* = 0.002], Time * average time spent on homework per week at 6 months [*F*(1; 100) = 5.3, *p* = 0.023], Time * baseline average amount of sleep [*F*(1; 100) = 7.4, *p* = 0.008], and finally Time * L cerebellum * R cerebellum GM volume changes [*F*(1; 100) = 9.6, *p* = 0.013]. Effects of Group, left caudate GM volume change, right Rolandic operculum GM volume change, Site, T0 age, Total Intracranial Volune and their interaction with Time were not significant.

**Fig. 4:**
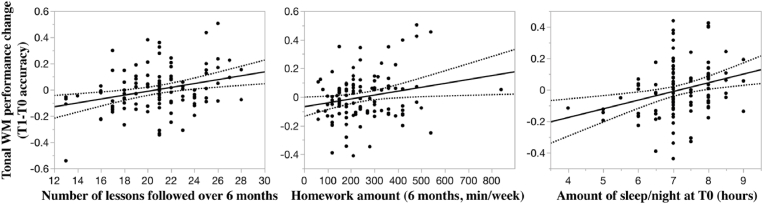
**The total number of lessons followed over 6 months, average time spent on homework per week at 6 months, and baseline average amount of sleep per night are positively associated with tonal working memory performance.** Post-hoc linear regressions. Individual tonal WM performance gains adjusted for the factors entered in the ANCOVA (residuals) except the factor on the x-axis (WM: working memory; min.: minutes).

The above-mentioned combination of significant factors explained 26% (R^2^ = 25.7%) of the variance of the tonal WM progress (post-hoc ANCOVA on T1-T0 tonal WM performance, F = 4.4, *p* < 0.0001). The total number of lessons followed over 6 months, the average time spent on homework per week at 6 months and the average amount of sleep per night at baseline predict independently and positively the gain of WM performance over 6 months. The larger the number of lessons followed over 6 months (R^2^ = 8.4, *p* = 0.002), time spent on homework at 6 months (R^2^ = 4.5, *p* = 0.03) or average amount of sleep at baseline (R^2^ = 7.9, *p* = 0.003), the larger the tonal WM performance improvement (Fig. 4).

**Fig. 5:**
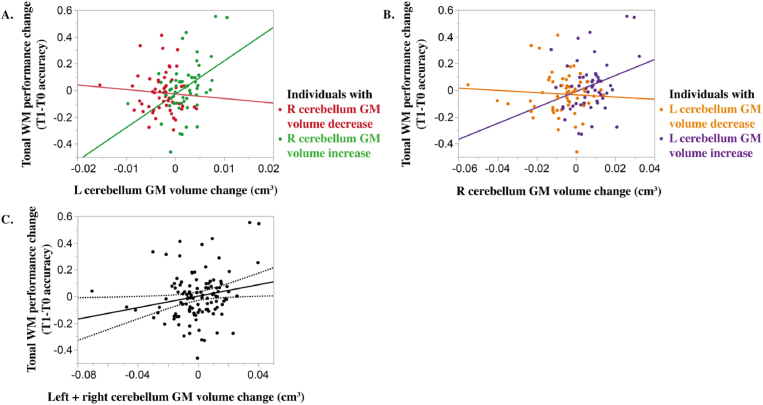
**Tonal working memory change is affected by the left and right cerebellar grey matter volume change.** Post-hoc linear regression. A. Performance change as a function of left cerebellum volume change is significant only in participants showing an increase of right cerebellum GM volume (left graph, participants color-coded in green, R^2^ = 19, *p* < 0.001). B. The linear regression between performance change and the right cerebellum volume change is only significant in participants with an increase of GM volume in the contralateral left cerebellum (right graph, participants color-coded in purple, R^2^ = 8.4, *p* < 0.04). C. Linear regression (R^2^ = 4, *p* < 0.04) between left and right cerebellum volume change (T1-T0 left cerebellum volume + T1-T0 right cerebellum volume) and tonal working memory performance change. Individual tonal working memory performance adjusted (residuals) for the factors entered in the ANCOVA except for the corresponding cerebellar volumes. Cerebellar volumes adjusted for total intracranial volume (WM: working memory, T0: baseline, T1: 6 months, L: left, R: right, GM: grey matter). (For interpretation of the references to color in this figure legend, the reader is referred to the Web version of this article.)

The GM volume changes of the left and right cerebellum ROIs interact (triple interaction Time*L cerebellum*R cerebellum GM volume change). We report a positive linear relationship between the left cerebellar GM volume increase and the tonal WM performance change in participants with a right cerebellar increase of GM volume (Fig. 5, N = 57, R^2^ = 19%, *p* < 0.001). This regression is not significant in participants showing a left cerebellar decrease of GM volume (N = 55, R^2^ = 0.6%, *p* = 0.59). Similarly, such a relationship between performance change and right cerebellar GM volume increase is present in participants with a left cerebellar increase of GM volume (Fig. 5, N = 54, R^2^ = 8.4%, *p* < 0.04). This relationship does not appear in the category of participants with a right cerebellar GM volume decrease (N = 58, R^2^ = 0.4%, *p* = 0.63). Hence the increase of contralateral cerebellar GM volume influences the relationship between its ipsilateral counterpart and the tonal WM performance change. Only a common pattern of cerebellar GM volume increase on both left and right hemispheres relates to a gain in performance. Atrophy on either the left or the right cerebellum clusters over 6 months is not associated with a change in performance.

Finally, we evaluated whether the total GM volume change in the left and right cerebellum (sum of the left and right volume change over time: T1-T0 left cerebellum volume + T1-T0 right cerebellum volume) linearly predicted tonal WM performance progress. The linear regression is significant (Fig. 5, R^2^ = 4%, *p* < 0.04). The larger the increase in cerebellar GM volume – both sides confounded, the larger is the tonal WM performance change.

#### 4.4.2 Verbal working memory improvement do not relate to plasticity or training intensity

The repeated-measures 2x2 ANCOVA full model did not detect significant effect of Time on Backward Digit Span performance. However, the Wilcoxon Signed-Ranked test as compared to 0 showed a significant increase in Digit Span Backward performance over 6 months both groups combined (T1 – T0 digit span backward performance mean in all participants = 0.39 ± 1.9, *W* = 1061, *p* = 0.02 two-tail signed-ranked test). This increase in performance is also significant in each group separately (mean P group performance = 0.39 ± 1.9, *W* = 301, *p* < 0.05 one-tail signed-ranked test; mean MC group performance = 0.38 ± 1.9, *W* = 230, *p* < 0.05 one-tail signed-ranked test).

The ANCOVA model also reveals significant main effects of Total Intracranial Volume at baseline [*F*(1; 106) = 9.3, *p* = 0.003], Site [*F*(1; 106) = 4.4, *p* = 0.04], and T1 – T0 right cerebellum volume change [*F*(1; 106) = 4.7, *p* = 0.03]. Post-hoc tests show that Total Intracranial Volume (Spearman's *ρ* = 0.4, *p* < 0.0001) was positively associated with better scores at both timepoints, grey volume plasticity of the right cerebellum did not. German participants performed better at both timepoints than Swiss participants (mean T0 + T1 Digit Span Backward score: German participants = 17.7 ± 3.7, Swiss participants = 15.4 ± 3.2, *Z =* −3.5, *p* < 0.0006). Age, training intensity, sleep, GM volume plasticity and group effects were not significant.

### 4.5 Local group differences in grey matter volume: a priori hypothesis testing

#### 4.5.1 Primary auditory TE1.0 area grey matter volume stays stable over time in the piano group, while it decreases in the musical culture group

**Fig. 6:**
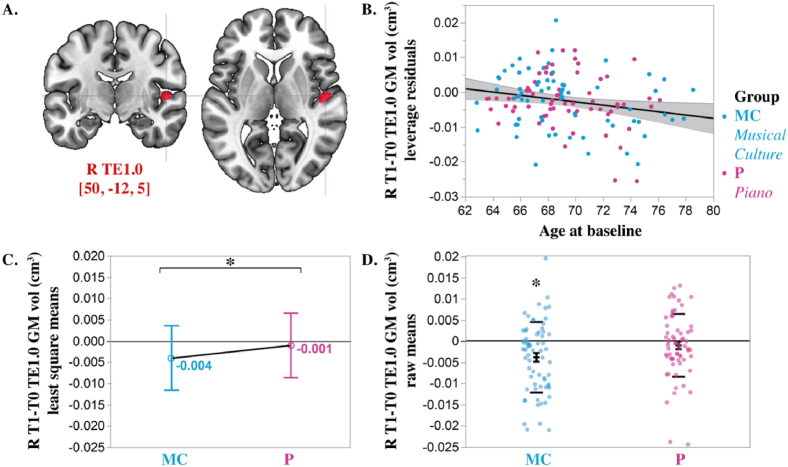
**Relationships between right TE1.0 primary auditory cortex volume change over time, Age and group factor.** A. Righht TE1.0 region (red) overlayed on MNI152 brain template (MNI coordinates of the ROI center are provided in mm). The right TE1.0 overlaps with the center of the right Heschl's gyrus. B. Negative relationship between the grey matter volume change of the right TE1.0 area and age at baseline. Group is color-coded (blue: musical culture group; pink: piano group). C. Group difference (*: *p* = 0.026) of the right TE1.0 grey matter volume change over 6 months (ANCOVA least square means plot). D. Grey matter volume change raw mean in the right TE1.0 area. The asterisk illustrates the significant grey matter volume decrease observed in the musical culture group (*: *p* = 0.0007, two-tailed Wilcoxon Signed-Rank test, *Z* = −514., *p* = 0.0007). (For interpretation of the references to color in this figure legend, the reader is referred to the Web version of this article.)

Results from the repeated-measure ANCOVA models evaluating the 6 TE1 areas GM volume changes over time (left T1-T0 TE1.0, left T1-T0 TE1.1, left T1-T0 TE1.2, right T1-T0 TE1.0, right T1-T0 TE1.1, right T1-T0 TE1.2) showed a significant main effect of Age [*F*(1; 124) = 4.9, *p* = 0.03] and a trend for an interaction Group * TE1 GM volume change [*F*(1; 124) = 3.2, *p* = 0.043]. This trend indicates that there might be a group difference in one or several TE1 subregions. Following the ANCOVA Least Squares Means plot indicating that this trend originated from the right TE1.0 area, an additional post-hoc ANCOVA model was performed to evaluate the right T1-T0 TE1.0 change between groups. Age [*F*(1; 126) = 6.0, *p* = 0.016] and Group effects were significant [*F*(1; 126) = 5.5, *p* = 0.026]. Age was negatively associated with the right TE1.0 GM volume. In other words, the higher the age, the higher the GM volume loss or atrophy. The group effect corresponded to a significant difference in GM plasticity in favor of the piano group (Fig. 6). While the right TE1.0 GM volume stayed stable over 6 months in the P group (least square mean = −0.001 ± 0.0078), the right TE1.0 GM volume decreased in the MC group (least square mean = −0.004 ± 0.0078). Post hoc testing of the raw mean as compared to 0 reveals indeed a stability of the GM volume over time in the P group (T1-T0 R TE1.0 GM vol = - 0.001 ± 0.0074, two-tailed *t*-test, *t* = −1.1, *p* = 0.27) as compared to a significant decrease in the MC group (T1-T0 R TE1.0 GM vol = −0.004 ± 0.0083, two-tailed Wilcoxon Signed-Rank test, *Z* = −514.5, *p* = 0.0007). However, training intensity metrics did not contribute to the right TE1.0 volume change over time (Number of lessons followed over 6 months and average time spent on homework per week at 6 months). In addition, the right T1-T0 TE1.0 GM volume change did not relate to variations in auditory WM performance.

### 4.6 Whole-brain grey matter volume decrease group differences (atrophy)

Group comparisons (two-sample *t*-test, P > MC and T0 > T1; MC > P and T0 > T1) did not reveal any significant statistical differences in atrophy between both groups over 6 months of training (*p* < 0.001, uncorrected, k = 20 voxels).

### 4.7 Whole-brain grey matter atrophy pattern over 6 months

**Fig. 7:**
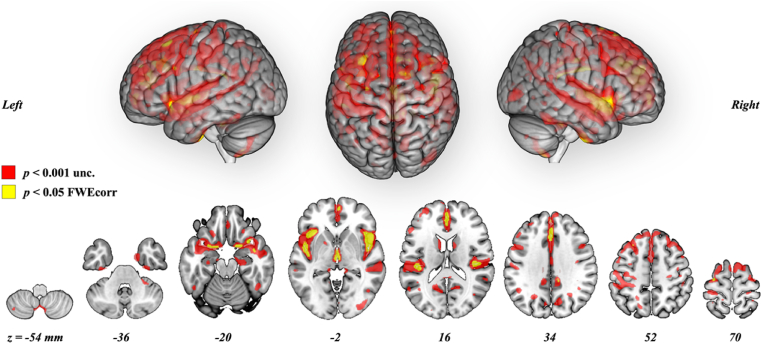
**Grey matter volume atrophy over 6 months in 132 individuals following music interventions (T0 > T1).** 3D render and ventral to dorsal locations of the axial slices are provided with z coordinate (MNI space). Results are displayed on MNI152 brain template with an extent threshold of *k* = 20 voxels and both statistical thresholds of *p* < 0.001 uncorrected for multiple comparisons (in red) and *p* < 0.05 FWE corrected (in yellow). (For interpretation of the references to color in this figure legend, the reader is referred to the Web version of this article.)

The whole-brain analysis of GM volume decrease conducted in all participants, both music training groups combined, shows a widely distributed fronto-parieto-temporal pattern of atrophy over 6 months (Fig. 7, Supplementary Table 2, *p* < 0.001 uncorrected, k = 20 voxels, one-sample *t*-test) including the neocortex, hippocampi and other subcortical structures, as well as the cerebellum. From the extraction of the mean total GM atrophy volume (−1.007 ± 1.207 cm^3^), we report a decrease of −0.17% in GM volume over 6 months (baseline GM volume = 589.139 ± 52.682 cm^3^). As expected, the pattern of atrophy was more restricted at *p* < 0.05 FWE corrected, with no significant clusters in the occipital cortex (Fig. 7, Table 3, k = 20 voxels).

**Table 3:** **Locations of clusters at peak voxels and statistics associated with grey matter volume decreases detected over 6 months of training in all individuals at *p* < 0.05 Family-wise error corrected (N = 132, k = 20 voxels).** MNI: Montreal Neurological Institute, ant.: anterior; post: posterior; L: left; R: right, inf: inferior; sup: superior; bil: bilateral; Mid: middle; IFG: inferior frontal gyrus; OFC: orbitrofrontal cortex; SMA: supplementary motor area; N = number.

Location of peak voxels	MNI coordinates (x, y, z)	Cluster size (N voxels)	T-value (peak-level)	FWEcorr *p*-value (peak-level)
Bil. Thalamus	0, −16, 3	347	8.25	*p* < 0.001
R Temporal Sup Gyrus	44, 9, −10	1814	8.03	*p* < 0.001
Bil. Frontal Sup Medial	0, 46, 28	575	7.51	*p* < 0.001
Bil. Inf. Cerebellum	4, −58, −63	123	7.32	*p* < 0.001
Bil. Post. Cingulate Gyrus	6, −14, 28	337	6.99	*p* < 0.001
L Mid. Frontal Gyrus	−33, 21, 58	129	6.87	*p* < 0.001
R Nucleus Accumbens	16, 4, −14	208	6.69	*p* < 0.001
L Temporal Sup Gyrus	−46, 0, −4	1283	6.55	*p* < 0.001
R Fusiform Gyrus	36, −10, 45	228	6.53	*p* < 0.001
Third Ventricule	0, 6, −9	81	6.46	*p* < 0.001
Bil. Ant. Cingulate Gyrus	0, 26, 34	409	6.41	*p* < 0.001
R SMA	12, 6, 74	32	6.22	*p* < 0.001
R cerebellum IV/V	21, −36, −32	44	6.19	*p* < 0.001
L Heschl's gyrus	−44, −27, 15	170	6.02	*p* = 0.001
R Post Cingulate Sulcus	10, −18, 39	84	5.99	*p* = 0.001
R SMA	8, 21, 68	52	5.83	*p* = 0.002
R Lingual Gyrus	10, −34, 2	20	5.80	*p* = 0.002
L Precentral Gyrus	−54, 12, 32	24	5.54	*p* = 0.005
R Post. Cingulate	12, −38, 36	24	5.52	*p* = 0.006
R OFC	3, 57, −2	100	5.44	*p* = 0.008
R Cerebellum VI	42, −48, −27	23	5.41	*p* = 0.009
L Frontal Sup	−27, −9, 70	22	5.31	*p* = 0.014

### 4.8 Grey matter atrophy pattern does not relate to auditory working memory

Further evaluations of the relationships between atrophy volume and change in WM performance over 6 months were not significant. Neither the significant total atrophy volume nor the volume of independent regions showing significant atrophy (Table 3, FWE corr, clusters > 300 voxels) did contribute to any of the tonal and verbal WM performance changes.

## 5 Discussion

### 5.1 Summary of the results

To the best of our knowledge, this is the first large-scale longitudinal investigation on GM volume plasticity and auditory WM changes following 6-month music interventions in musically naïve healthy older adults. In absence of a group effect at the level of whole brain structure or WM performance, both music training regimens were pooled together (N = 132 participants). For both groups combined, our results show GM volume increases in the left caudate nucleus, the right Rolandic operculum and in the lobules VIII of the left and right cerebellum after 6 months of piano and music sensitization lessons (musical culture). Importantly, the GM volume increase in the left and right inferior posterior cerebellum, as well as training intensity, and finally average sleep duration, were positively associated with a significant gain in tonal WM performance after six months of interventions, arguing against a mere test-retest WM improvement. Those results suggest the occurrence of a near transfer effect of music training on tonal WM brain and behavioral substrates. They support the critical role of sleep and regular sustained training for learning and WM. In addition, music interventions were associated with significant increase in verbal WM as evaluated by the Backward Digit Span, but it did not correlate with training intensity metrics or GM volume plasticity. We validated our hypothesis of a local GM volume group difference in favor of the piano group at the level of the primary auditory cortex, in the right TE1.0 area, a core primary auditory subfield. GM volume stayed stable in the piano group while it declined significantly in the musical culture group (experimental group > active control group). Yet this macrostructural difference did not relate to auditory WM outcomes. Finally, we took advantage of this design and this methodology to evaluate not only the positive effects of our intervention, but also detrimental effects of time, i.e., the GM volume atrophy occurring over 6 months despite training. A significant fronto-temporo-parietal GM volume atrophy pattern consistent with results from the literature was detected.

### 5.2 Grey matter volume increases in the elderly brain following music interventions

Both piano and musical culture groups combined, we show largely distributed GM volume increase (Fig. 3). Such a level of GM plasticity in the elderly brain is remarkable, confirming the plasticity potential of the aging brain contrary to old beliefs (Cespón et al., 2018; Intzandt et al., 2021; Ten Brinke et al., 2017). The here reported results strengthen the relevance of music training as a model for learning-driven brain plasticity (Altenmüller and Schlaug, 2015; James et al., 2014b; Jancke, 2009; Münte et al., 2002; Oechslin et al., 2013a; Oechslin et al., 2018; Oechslin et al., 2013c; Vuust et al., 2022; Wan and Schlaug, 2010; Zatorre et al., 2012). The current study extends these results to the framework of aging. Music training may buffer against some age-related cognitive decline effects, in accordance with other recent publications from our group (Jünemann et al., 2022; Worschech et al., 2022).

The increase of GM volume in the left caudate nucleus is particularly interesting because this region is involved in goal-directed behavior, and belongs to the executive system (Grahn et al., 2008). Piano and musical culture training both rely on such abilities. Playing music requires constant monitoring and analysis of the keys played to match the musical score or the auditory imagination to play a piece accurately. The musical culture intervention focused on analytical listening, supported by gradually increasing musical knowledge. For both interventions, associated GM plasticity of the left caudate nucleus may thus relate to executive function benefits (eg. inhibition or monitoring), particularly affected by aging (Buckner, 2004; Ferguson et al., 2021; Rozas et al., 2008).

The increase of GM volume in the right Rolandic/central operculum should be interpreted with caution because of the heteromodality of this cortex, involved in different cognitive processes such as language, and its widespread connectivity (Behroozmand et al., 2015). The operculum is considered as the largest sensory processing neurocircuit by some authors, with four distinct primary areas (auditory, taste, pain and vestibular), several secondary areas mainly involved in sensory auditory integration and a motor area for the oropharynx (Mălîia et al., 2018). The location of the cluster peak is central operculum. Single-pulse electrical stimulation of this region in epileptic patients is mostly associated with oropharyngeal responses (Mălîia et al., 2018). The cluster peak is overlapping with activations reported in the central operculum by Koelsch and colleagues during the perception of pleasant tunes in musically naive young adults (Koelsch et al., 2006). They interpreted the role of this region as part of a motor-related circuitry responsible for the formation of (premotor) representations for vocal sound production corresponding to perception of pleasant auditory information. Considering the large extent of the cluster on the full temporal, central and frontal operculum when results are not corrected for multiple comparisons, we argue that this GM volume increase centered on the right central operculum may relate to the stimulation of audio-motor coupling between music perception and covert vocal production during both musical activities.

### 5.3 Cerebellar grey matter and tonal working memory plasticity: a structure-behavior relationship

Last but not least, we report a GM structure-behavior relationship at the level of the cerebellum for our outcome of interest, auditory WM, in particular tonal WM as measured with a task performed in the noisy MRI scanner environment. Significant increases in GM volume were detected in the bilateral lobules VIII that extend into lobule IX. Overall participants’ performance improved 6% on average. Increase in cerebellar GM volumes positively correlated to tonal WM progress over 6 months. The right lobule VIII was previously associated with auditory motion, and the ability to detect a directional auditory motion signal in noise at various signal levels (Baumann and Mattingley, 2010), but also to timbre discrimination in a performance-dependent manner (Reiterer et al., 2008). Both discrimination of timbre (auditory characteristics allowing to distinguish sounds that have the same pitch and loudness), and of auditory motion signal from noise, are acoustically demanding tasks that require a fine level of sensory control and more importantly, auditory WM, implicated in the tonal WM task used in this study.

The longitudinal structure-behavior relationship we report at the level of the cerebellum and the WM is in accordance with reviews of neuroimaging studies (Guell and Schmahmann, 2020; Marvel and Desmond, 2010), revealing that the inferior cerebellar lobules VIII are involved in WM. The inferior cerebellar lobules are particularly active when information must be held in mind across a delay. They potentially support the phonological storage function or motor-related aspects of speech as part of WM such as comparing speech output (overt or covert), or tones in the case of this study, with information contained in online storage. In addition, our finding follows the results of a cross-sectional study conducted with a similar methodology in a mixed sample of young and older adults, where verbal WM accuracy was related to left posterior cerebellar volume including notably lobules VIII and IX, such that larger volume resulted in better performance (Marvel and Desmond, 2010). The cerebellum has been historically linked to the gross/fine motor control and skills. Yet, reports on the cognitive role of the cerebellum began to increase sharply in the early 90′s. Cerebellar damage was associated with impairment of many cognitive functions such as WM (verbal and spatial), executive function or language (Stoodley and Schmahmann, 2009). In line with those studies, our cerebellum GM volumetric finding, deriving from an exploratory whole-brain analysis corrected for multiple comparisons, and its relationship with auditory WM is important. The novelty of this result lies in the longitudinal aspect of this study and the framework of lifespan development and aging. Beyond neuroprotection, this finding calls for a positive transfer of music training to cerebellum substrates of auditory WM, associated with increased performance. This is relevant for healthy aging as both cerebellum volume and WM, are singularly affected by decline (Marvel and Desmond, 2010). Further investigation is needed to evaluate the impact of such plasticity on the auditory WM network. Nevertheless, this result supports the claim that stimulating cross-modal group interventions (eg. physical or music training) are promising approaches to support healthy aging (Intzandt et al., 2021). In this context, musical culture/active listening may be a promising low-cost ecological behavioral group intervention, simpler than piano or other instrumental interventions.

### 5.4 Music training transfers to auditory working memory

Numerous studies report enhanced auditory/visual WM skills in musicians as compared to non-musicians (Oechslin et al., 2013c; Schulze and Koelsch, 2012; Yurgil et al., 2020) also in healthy older adults (Bugos, 2019; Bugos et al., 2007; Bugos and Wang, 2022; Dege and Kerkovius, 2018). We show a significant tonal WM progress following 6-month music interventions in relation with training intensity metrics, i.e. the number of lessons followed by the participants over 6 months and the average time spent on homework at 6 months. The more the participants attended to lessons and practiced at home, the higher was their increase in tonal WM performance. These results call for a close transfer effect of music training on tonal WM. They suggest that frequency and intensity of training contribute to this transfer. We also report a significant increase in verbal WM performance in the Backward Digit Span task associated with the music interventions, however it did not correlate with training intensity metrics or cerebellar GM changes.

Such positive verbal WM result is expected given the history of musical training effects on verbal WM (Franklin et al., 2008; Pozenatto, 2020). Such transfer may represent a more distant form of behavioral transfer in the language executive function domain, even though music and language may rely to a certain degree on shared resources (James, 2012; Jäncke, 2012) notably for WM (Fennell et al., 2021). There is a debate coming from the musical training literature on the unity of auditory short-term and WM systems regarding tonal and verbal information (Salamé and Baddeley, 1989; Ueda, 2004; Williamson et al., 2010). Schultze and colleagues evaluated and reviewed the similarities and differences between verbal and tonal WM at the brain functional level (Schulze and Koelsch, 2012; Schulze et al., 2011). They describe a considerable overlap of neural resources underlying WM for both verbal and tonal information in non-musicians, but several regional differences between verbal and tonal WM networks in musicians. They suggested the presence of a tonal loop in addition to the phonological loop system in musicians. It is plausible that the increase in tonal WM performance found in this study may relate to the development of the tonal loop system, further investigation is needed at the brain functional level.

Beyond music expertise, our auditory WM results must also be discussed in the framework of longitudinal investigations on music training in healthy older adults, mostly showing enhanced visual WM following music interventions (Bugos, 2019; Bugos et al., 2007; Bugos and Wang, 2022; Degé and Kerkovius, 2018)(Seinfeld et al., 2013). This discussion is problematic for methodological reasons. Visual WM evaluations were mostly embedded in tests evaluating other domains as well (eg. Stroop task and inhibition). None of these studies assessed tonal WM, and only two studies evaluated verbal WM (Dege and Kerkovius, 2018; Seinfeld et al., 2013). In addition, these studies did not document training intensity. Randomization was not always applied, and mean sample size was 2.5 times lower than in the current study considering the experimental group, which may have led to sample size issues. In contradiction with our positive verbal WM results, two studies included the Digit Span as well but reported no group difference (Dege and Kerkovius, 2018; Seinfeld et al., 2013). Yet those studies included a number of participants ranging from 8 to 13 in the music group, training duration lasted 15–16 weeks, participants were not requested to practice at home in one study (Dege and Kerkovius, 2018), while the instrument was not provided for home practice in the other (Seinfeld et al., 2013). Thus, it is possible that music training transfer effects were limited in these studies.

Further investigations are needed to conclude on the transfer effects of music training on auditory WM across healthy aging studies and in our population. The tonal and verbal WM improvement following music training observed in the current study is very encouraging. Yet as the verbal WM progress did not relate to training intensity, we must acknowledge the possibility of test-retest effect. Further analysis of our 12 months data may shed further light on the issue. We expect maintenance of the musical training effects on tonal and verbal WM in association with training intensity metrics, and a potential group difference in favor of the piano group that receives a more specialized form of auditory WM training than the musical culture group. Nevertheless, the WM improvement observed here after 6 months of music intervention is already promising, WM being involved in many aspects of cognitive functioning and activities of daily living (Cantarella et al., 2017; McDougall et al., 2019).

### 5.5 The neuroprotective effect of piano learning on the core of the right primary auditory cortex

In absence of 6-month whole-brain group differences between intervention groups (piano and musical culture), we tested the hypothesis of a local group difference in favor of the piano group at the level of the primary auditory cortex, the first cortical sound processing level, located in Heschl's gyrus. This region was selected because it is widely affected by aging and strongly implicated in musical activities (Crivello et al., 2014; Jiang et al., 2014; Profant et al., 2014). This hypothesis was also based on numerous reports of larger GM volume in Heschl's gyri in musicians particularly (Bermudez et al., 2009; Dalboni da Rocha et al., 2020; Gaser and Schlaug, 2003; James et al., 2014a; Schneider et al., 2002), and two recent studies from our group on the same population. We showed a group difference in favor of the piano group at the level of the left acoustic radiation fiber bundle cross-section (Jünemann et al., 2022), and cortical thickness of the bilateral auditory region (Worschech et al., 2022). In addition, the latter study reported positive relationships between cortical thickness of the right Heschl's gyrus and speech in noise perception. Given the causal role of the auditory cortex in auditory WM (Kumar et al., 2016; Yu et al., 2021), we expected that the presence of a group difference in the primary auditory cortex GM volume might relate to auditory WM differences.

We show a stabilization or maintenance of GM volume in one of the three primary auditory cortex subfields on the right side, the right TE1.0 area, in the piano group as compared to a decline in the musical culture group. Interestingly, among the three examined subregions, TE1.0 is considered as the koniocortical core field because it includes the widest granular layer IV and so potentially, the biggest thalamic input (Morosan et al., 2001). Interestingly TE1.0 area, the koniocortical primary core field as defined by cytoarchitectonics, overlaps with the primary auditory cortex identification from cortical thickness and primary source auditory activity features (Zoellner et al., 2019). In the context of aging, a stabilization at the level of the primary auditory cortex core is a positive result that integrates nicely with our previous reports of piano benefits on the central auditory system in the same population (Jünemann et al., 2022; Worschech et al., 2022). However, this macrostructural primary auditory cortex protection against aging conveyed by piano training did not relate to auditory WM improvement or maintenance. Considering the right hemisphere specialization for tonal and spectral processing and its relationship with Heschl's gyrus macroanatomy (Schneider et al., 2005; Warrier et al., 2009; Zatorre and Belin, 2001; Zatorre et al., 2002), we postulate that this GM maintenance plasticity effect associated with piano training may relate to audio-motor coupling, sound to object associations (tone to keyboard key), pitch processing and/or ear training. This observation is in accordance with a recent study showing increased right primary auditory evoked fields and auditory skills following musical active listening training (Schneider et al., 2022). Unfortunately, we did not acquire material to test such a hypothesis beyond the tonal WM task.

### 5.6 6-month grey matter atrophy is evident in normal aging

Beyond the music training's neuroprotective and near transfer effects associated with brain and behavioral plasticity, we report a significant fronto-temporo-parietal GM volume atrophy pattern. We took advantage of this longitudinal randomized controlled trial design, conducted in participants carefully selected for good health, and the longitudinal VBM method to evaluate the detrimental effects of normal aging over 6 months in all 132 individuals. The current study is the first interventional study reporting such detrimental effects over only 6 months despite stimulating activities. It is important to acknowledge that brain physiological decline is inevitable, yet it does not prevent local positive brain plasticity following stimulating and adaptive learning interventions.

The aging brain undergoes significant atrophy despite intense cognitive training, here music, in healthy older adults. Those atrophy effects are stronger in terms of statistical power (Cohen's *d* = T/√(degree of freedom), *d* = 0.73 for atrophy vs. 0.6 for GM volume increase for the most significant cluster) and extent (19′462 for atrophy vs. 1′952 voxels for GM volume increase, most significant cluster) than plasticity effects associated with cognitive training. We report an average total GM volume decrease of 1′007 mm^3^; 0.17% of the baseline GM volume. This is in line with the 2′497.5 mm^3^ of GM volume loss reported over 1 year in 228 healthy older adults (Bagarinao et al., 2022). The significant fronto-temporo-parietal GM volume atrophy pattern that we show is consistent with cross-sectional voxel-based morphometry comparison between young and older adults (Morand et al., 2020) and more importantly, the longitudinal whole-brain morphometric literature. According to Fjell and colleagues, one year is enough to detect a global significant atrophy distributed in an anterio-posterior gradient, with fast rates of decline in the frontal and temporal-parietal cortex as compared to the relatively preserved occipital cortex (Bagarinao et al., 2022; Fjell and Walhovd, 2010; Fjell et al., 2009; Jiang et al., 2014). Actually, according to our results, 6 months may be sufficient to detect whole-brain atrophy corrected for multiple comparisons, if the sample is large and the design is longitudinal.

From the review of this literature, we note that the longer the longitudinal time window, the stronger is the atrophy pattern and its potential extension to the occipital cortex. We may expect such results to appear in our future analyses over 1.5 years. We can assume that regions detected by our 6-months analysis show a fast rate of decline. Beyond the 6 largest and most significant atrophy clusters (>300 voxels, bilateral thalami, left and right temporal superior gyri, bilateral medial superior frontal gyri, bilateral anterior and posterior cingulate gyri), for which we suspect potential relationships with cognitive decline, we report atrophy in regions classically described in the literature such as the frontal cortex, hippocampi, basal ganglia and cerebellum subregions (Fjell and Walhovd, 2010; Fraser et al., 2015; Hicks et al., 2022; Jiang et al., 2014), but also other regions less frequently reported like the left Heschl's gyrus (Crivello et al., 2014; Jiang et al., 2014; Profant et al., 2014). Interestingly, the global and regional patterns of atrophy did not relate to auditory WM performance. The fact that such atrophy, notably in the auditory cortex, was not associated with behavioral decline demonstrates the resilience of brain functions and associated behavior to atrophy, suggesting cognitive reserve (Cabeza et al., 2018) and ways to strengthen it are a key for healthy aging (Stern, 2009). We postulate that the music interventions compensated for loss in WM performance, but further investigation is needed.

### 5.7 Conclusions

Overall, global and local GM changes did not relate to musical training intensity metrics per se. But GM volume increase of the inferior cerebellum and training intensity were independently associated with tonal WM performance change, suggesting an indirect link between GM volume plasticity, musical training, and near behavioral transfer. Interestingly musical training was also associated with verbal WM benefits (Backward Digit Span), a more distant form of transfer than tonal WM. Piano training was associated with neuroprotection of the primary auditory cortex against atrophy, but it did not relate to training intensity metrics. Musical training transfer effect mechanisms on GM volume plasticity were thus not completely elucidated. It is possible that we cannot relate a capture of GM volume plasticity dynamics with training intensity metrics averaged over a large time window of 6 months. In addition to the 6-month music intervention positive effects, we also show largely distributed atrophy. Brain and auditory WM decline is inevitable, but brain structure and behavior remain plastic in older adults. Based on our results, we argue that education for seniors should become a major policy priority in the framework of healthy aging, to promote brain plasticity, cognitive reserve, mental health, independence, and well-being through stimulating, cross-modal, group interventions such as musical interventions.

The neuroanatomical and behavioral findings presented in this study demonstrate that music interventions can mitigate cognitive decline. Our research team previously showed similar findings evaluating other brain features and behavioral aspects (Jünemann et al., 2022; Worschech et al., 2021, 2022). Other authors’ behavioral results confirm these findings (Bugos, 2018; Galinha et al., 2020; Rouse et al., 2022; Schneider et al., 2018). It was already known that lifelong musical instrumental practice protects to some extent against cognitive decline and dementia (Balbag et al., 2014; Gooding et al., 2014; Verghese et al., 2003), potentially through its contribution to cognitive reserve (Cabeza et al., 2018). Taken together, these results suggest that not only music education - music practice, active listening and possibly singing (Flo et al., 2022), can countervail cognitive decline and dementia and promote brain preservation in later life, but also that stimulating activities at a younger age may be beneficial, including, for example, physical exercise (Gaertner et al., 2018). So, prevention of cognitive decline should start way before getting old.

## Author contributions

DM: conceptualization, investigation, data curation, formal analysis, and writing—original draft preparation. CAHM: data preprocessing and analysis—review and editing. FW: conceptualization, investigation, and writing—review and editing. DS, FG, and DV: conceptualization and writing—review and editing. MK: detailed input on funding acquisition, conceptualization, and writing—review and editing. CJ: funding acquisition, conceptualization, supervision, and writing—review and editing. EA: funding acquisition, conceptualization, supervision, review and editing. TK: detailed input on funding acquisition, conceptualization, supervision, review, and editing. CS: conceptualization, supervision, review, and editing. All authors contributed to the article and approved the submitted version.

## Ethical approval and informed consent

The randomized control trial protocol approved by the Ethics Committee of Geneva (no. 2016–02224) and the Ethikkomission of the Leibniz Universität Hannover (no. 3604–2017) was conducted in accordance with Helsinki Declaration. Informed consent was obtained from all patients (James et al., 2020a). The full protocol was registered at clinicaltrials.gov (NCT03674931, no. 81185).

## Funding

This work was funded by the 10.13039/501100001711Swiss National Science Foundation (grant no. 100019E-170410) and the 10.13039/501100001659German Research Foundation (grant no. 323965454). Financial support was also provided by the Med. Kurt Fries Foundation, the Dalle Molle Foundation, and the Edith Maryon Foundation.

## Disclosure statement

The authors report no competing interests.

## Declaration of competing interest

The authors declare that they have no known competing financial interests or personal relationships that could have appeared to influence the work reported in this paper.

## Data Availability

Data will be made available on request.
