# Supplementary material for: Music interventions in 132 healthy older adults enhance cerebellar grey matter and auditory working memory, despite general brain atrophy
Source: Neuroimage Rep. 2023 Mar 23;3(2):100166. doi: 10.1016/j.ynirp.2023.100166 (PMC12172798; doi:10.1016/j.ynirp.2023.100166)
Supplement: Multimedia component 1 [file mmc1.docx]

**Supplementary Table 1. Locations of clusters at peak voxels and statistics associated with grey matter volume increases detected over 6 months of music training in all individuals at a statistical threshold of *p* < 0.001 uncorrected for multiple comparisons (N = 132, k = 20 voxels).** L: left; R: right, inf: inferior; sup: superior; mid: middle; MNI: Montreal Neurological Institute; N = number.

| **Location of peak voxels** | **MNI coordinates (x, y, z)** | **Cluster size (N voxels)** | **T-value (peak-level)** |
| --- | --- | --- | --- |
| L and R inf. cerebellum | 15, -52, -45 | 1952 | 6.79 |
| L caudate nucleus | -20, 10, 18 | 261 | 5.66 |
| R rolandic operculum | 46, -2, 16 | 1329 | 5.49 |
| R sup. cerebellum | 10, -45, -14 | 235 | 5.16 |
| R inf. temporal gyrus | 39, -4, -30 | 331 | 4.98 |
| R thalamus | 20, -21, 14 | 79 | 4.43 |
| L precentral gyrus | -40, -2, 38 | 78 | 4.27 |
| L rolandic operculum | -36, -33, 24 | 32 | 4.05 |
| R hippocampus | 30, -26, -6 | 27 | 4.03 |
| L mid. cingulate gyrus | -12, 26, 30 | 52 | 3.52 |
