# Supplementary material for: Music interventions in 132 healthy older adults enhance cerebellar grey matter and auditory working memory, despite general brain atrophy
Source: Neuroimage Rep. 2023 Mar 23;3(2):100166. doi: 10.1016/j.ynirp.2023.100166 (PMC12172798; doi:10.1016/j.ynirp.2023.100166)
Supplement: Multimedia component 2 [file mmc2.docx]

**Supplementary Table 2. Locations of clusters at peak voxels and statistics for grey matter volume decrease detected over 6 months at a statistical threshold of *p* < 0.001 uncorrected for multiple comparisons (N = 132, k = 20 voxels).** MNI: Montreal Neurological Institute, ant.: anterior; post: posterior; L: left; R: right, inf: inferior; sup: superior; bil: bilateral; Mid: middle; IFG: inferior frontal gyrus; OFC: orbitofrontal cortex; orb.: orbitalis; SMG: supramarginal gyrus; SMA: supplementary motor area; N = number.

| **Location of peak voxels** | **MNI coordinates (x, y, z)** | **Cluster size (N voxels)** | **T-value (peak-level)** |
| --- | --- | --- | --- |
| Bil. Thalamus | 0, -16, 2 | 19462 | 8.25 |
| Bil. Frontal Sup. Medial | 0, 46, 28 | 17521 | 7.51 |
| Bil. Cerebellum VII / IX | 4, -58, -63 | 681 | 7.32 |
| R Fusiform Gyrus | 36, -10, -45 | 1891 | 6.53 |
| R Ant. Lingual Gyrus | 10, -34, 2 | 120 | 5.80 |
| L Post. Cingulate Gyrus | -12, -42, 33 | 180 | 5.63 |
| L Mid. Cingulate Gyrus | -10, -20, 38 | 202 | 5.42 |
| L Mid. Occipital Gyrus | -26, -63, 33 | 176 | 5.42 |
| R Cerebellum Crus I | 42, -48, -27 | 498 | 5.41 |
| L Post/Precentral Gyrus | -50, -15, 57 | 3440 | 5.26 |
| L Calcarine | -18, -57, 16 | 875 | 5.26 |
| L Caudate Nucleus | -8, 3, 14 | 298 | 5.06 |
| R Precentral/inf. Frontal Sulcus Junction | 51, 12, 27 | 1051 | 4.97 |
| R Occipital Inf. Gyrus | 36, -87, -2 | 548 | 4.70 |
| R Frontal Sup. | 27, -6, 69 | 170 | 4.66 |
| R Caudate Nucleus | 9, 4, 12 | 233 | 4.64 |
| L Post. OFC | -20, 21, -22 | 257 | 4.61 |
| L Angular Gyrus | -42, -52, 22 | 405 | 4.60 |
| R Cuneus | 14, -70, 32 | 585 | 4.54 |
| L Ant. Calcarine Sulcus | -12, -54, 3 | 185 | 4.48 |
| R Precentral Gyrus | 38, -9, 45 | 309 | 4.26 |
| R Occipital Sup. | 27, -58, 36 | 153 | 4.24 |
| R Precentral Gyrus | 26, -4, 48 | 72 | 4.22 |
| R Precentral Gyrus | 27, -27, 51 | 52 | 4.18 |
| L Sup. Parietal Lobule | -30, -44, 66 | 69 | 4.17 |
| R Postcentral Gyrus | 42, -24, 40 | 317 | 4.08 |
| L Cerebellum IV/V | -8, -46, -24 | 96 | 4.03 |
| L Occipital Mid. Gyrus | -22, -90, 14 | 52 | 3.93 |
| L Cerebellum Crus 2 | -39, -69, -46 | 180 | 3.91 |
| L Temporal Mid. Sulcus | -57, -58, 14 | 330 | 3.90 |
| Bil. Calcarine | 2, -81, 10 | 87 | 3.89 |
| L Mid. Frontal Gyrus | -32, -3, 48 | 103 | 3.84 |
| L Fusiform Gyrus | -36, -20, -38 | 94 | 3.83 |
| L Post. Temporal Inf. Gyrus | -48, -54, -21 | 54 | 3.79 |
| L IFG pars Orb. | -48, 40, -9 | 45 | 3.74 |
| R SMA | 8, -8, 76 | 27 | 3.73 |
| R Temporal Mid. Gyrus | 60, -52, -8 | 54 | 3.72 |
| R Postcentral Sulcus | 48, -33, 56 | 75 | 3.72 |
| R Central Sulcus | 24, -28, 74 | 88 | 3.71 |
| L Hippocampus | -14, -12, 22 | 52 | 3.69 |
| R Temporal Mid. Gyrus | 46, -64, -2 | 34 | 3.68 |
| R Occipital Mid. Gyrus | 30, -72, 24 | 104 | 3.68 |
| R Temporal Mid. Gyrus | 64, -38, -15 | 41 | 3.66 |
| R SMG | 39, -39, 38 | 53 | 3.65 |
| R Occipital Inf. Gyrus | 44, -75, -12 | 52 | 3.63 |
| R Cerebellum Crus II | 34, -74, -44 | 49 | 3.63 |
| L Angular Gyrus | -39, -64, 39 | 35 | 3.63 |
| L Temporal Mid. Gyrus | -40, -62, 16 | 25 | 3.61 |
| R Hippocampus | 38, -33, -6 | 39 | 3.59 |
| R Postcentral Gyrus | 32, -40, 68 | 32 | 3.54 |
| L Calcarine | 3, -82, -6 | 31 | 3.52 |
| R Angular Gyrus | 42, -51, 34 | 25 | 3.51 |
| L Inf. Occipital Gyrus | -46, -68, -9 | 21 | 3.46 |
